# Widespread and Heterologous Effects of L-DOPA on Monoaminergic Tissue Metabolism in Newborn Rats Expressing Air-Stepping

**DOI:** 10.3390/ijms26052298

**Published:** 2025-03-05

**Authors:** Grégory Barrière, Zora Pelloquin-Mvogo, Marie Boulain, Inès Khsime, Rahul Bharatiya, Marie-Anne Riquier, Didier Morin, Anne-Emilie Allain, Abdeslam Chagraoui, Laurent Juvin, Philippe De Deurwaerdere

**Affiliations:** 1 Institut de Neurosciences Cognitives et Intégratives d’Aquitaine, UMR5287, Centre National de la Recherche Scientifique, Bordeaux Neurocampus Department, University of Bordeaux, 33000 Bordeaux, France; gregory.barriere@u-bordeaux.fr (G.B.); zorapm33@yahoo.fr (Z.P.-M.); marie.boulain@outlook.fr (M.B.); khsime.ines@gmail.com (I.K.); bhartiyarahul20@gmail.com (R.B.); marida13240@gmail.com (M.-A.R.); didier.morin@u-bordeaux.fr (D.M.); anne-emilie.allain@u-bordeaux.fr (A.-E.A.); laurent.juvin@u-bordeaux.fr (L.J.); 2 Neuronal and Neuroendocrine Differentiation and Communication Laboratory, Institute for Research and Innovation in Biomedicine of Normandy (IRIB), Institut National de la Santé et de la Recherche Médicale U1239, University of Rouen Normandy, 76000 Rouen, France; abdeslam.chagraoui@univ-rouen.fr; 3 Department of Medical Biochemistry, Centre Hospitalo-Universitaire de Rouen, 76000 Rouen, France

**Keywords:** monoamine, neuromodulation, neurochemistry, central nervous system, locomotion, neonatal rat, high-pressure liquid chromatography, connectivity

## Abstract

L-DOPA triggers a dose-dependent increase in locomotor activity in newborn rats suspended in the air (air-stepping). Here, we report the effects of L-DOPA injection on the tissue level of monoamines and metabolites in different regions of the central nervous system (CNS) of postnatal day 5 pups. We also established correlations between some of our neurochemical measurements and basic locomotor parameters. L-DOPA (25–100 mg/kg) enhanced its tissue levels in the spinal cord, cortex, striatum, and brainstem regions. It induced a strong increase in the levels of the L-DOPA, dopamine, and their metabolites but had low effects on noradrenaline and serotonin across CNS regions. Of note, we also detected the tyramine derivative octopamine in the spinal cord. The inter-regional pattern of correlations between monoamine content showed an almost full metabolic connectivity for dopamine only when all L-DOPA conditions were pooled, and it revealed restricted connectivity for noradrenaline and serotonin in the spinal cord and the mesencephalic locomotor region. Locomotor parameters (quadrupedal locomotion and step numbers) correlated with the levels of L-DOPA and DA in restricted CNS regions at variance with noradrenaline and serotonin. Altogether, our data extend the idea that the neurochemical effect of L-DOPA is widespread and heterogeneous in the CNS, with prominent biochemical changes notably present in the spinal cord and M1 cortex, to the newborn rat.

## 1. Introduction

L-DOPA has been used in the treatment of Parkinson’s disease since the beginning of the 1960s, just after the discovery of tissue dopamine (DA) deficiency in the caudate nucleus/putamen (striatum in rodents) of deceased Parkinson’s patients [1,2]. It remains one of the best therapeutic approaches to the disease to date [3]. The neurobiological effects of L-DOPA are thought to be consequent to its decarboxylation into DA. However, beyond the fact that the side effects are dramatic after a few years of treatment [4], a growing amount of evidence has challenged the initial belief that L-DOPA’s therapeutic effects are simply due to its decarboxylation to DA in the striatum [5,6]. Yet, its mechanism of action is still misunderstood.

“The magic L-DOPA” [3] not only suits the treatment of Parkinson’s disease but also numerous preclinical findings that are relevant to spinal locomotor program. For years, L-DOPA has been shown to activate locomotion in different species, at different developmental stages, and under various conditions [7,8,9,10,11,12]. In the neonatal rat, L-DOPA triggers a dose-dependent activation of the locomotor program expressed when the animal is suspended in the air (air-stepping activity, [13,14,15,16,17]). L-DOPA increased the number of steps and time spent walking, likely by reducing the pause in the air-stepping model [13,18], in line with electromyographic data [10], but did not act on the frequency of steps [13,18]. This behavioral response is presumably dependent on dopaminergic responses in this model [19] and is likely related to massive neurochemical effects at the level of the spinal cord [13,20], but a contribution of supraspinal effects has been also previously proposed [21].

Exogenous L-DOPA exerts complex metabolic effects involving at least the enzymes aromatic amino acid decarboxylase (AADC) to produce DA and catechol-O-methyl transferase (COMT) to produce 3-O-methyl-DOPA (3-OMD). It also interferes with the metabolism of tyrosine, which could lead to additional trace amines [5,22]. The rise in L-DOPA-derived DA subsequently mobilizes the monoamine oxidases (MAOs) and COMT. These enzymes are not selective for DA neurons and are distributed along the central nervous system (CNS, [5]). Finally, L-DOPA disturbs the biochemical activity of serotonergic and noradrenergic terminals, which are widespread [5,23,24,25]. It implies that the effects of exogenous L-DOPA on monoaminergic compounds and metabolites occur virtually in the whole CNS, but whether this is the case in newborn rats exhibiting episodes of air-stepping remains unknown.

**Figure 1:**
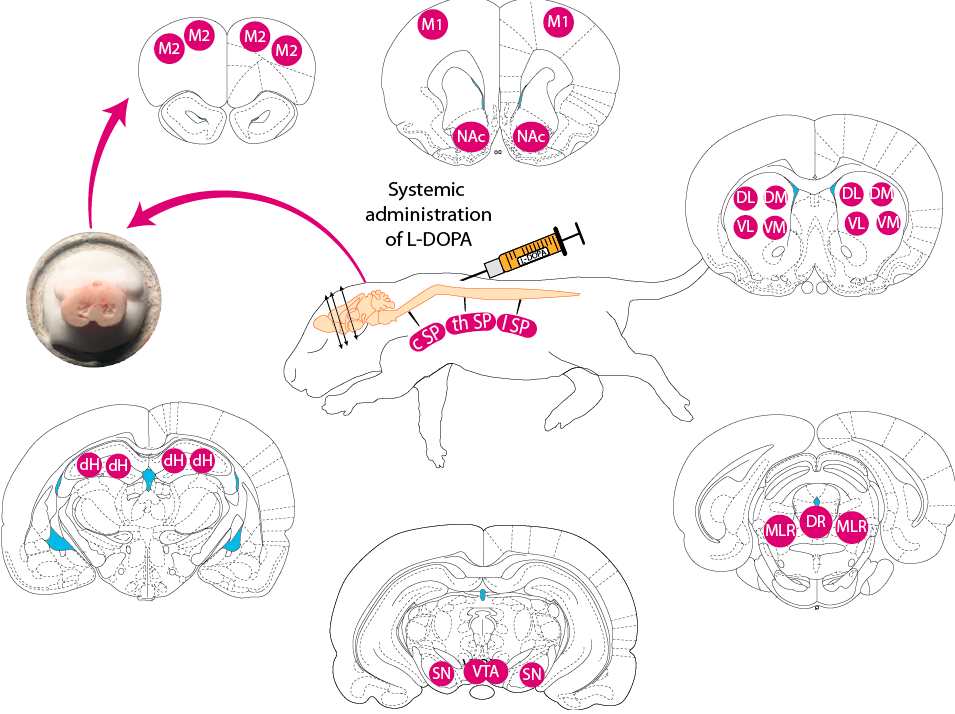
CNS regions sampled in PND5 newborn rats. cSP, cervical spinal cord; thSP, thoracic spinal cord; lSP, lumbar spinal cord; M1, M2, motor cortex; nA, nucleus accumbens (NAc); DM, dorsomedial striatum; DL, dorsolateral striatum; VL, ventrolateral striatum; VM, ventromedial striatum; dH, dorsal hippocampus; SN, substantia nigra; VTA, ventral tegmental area; MLR, mesencephalic locomotor region; DR, dorsal raphe. L-DOPA or its vehicle was administered peripherally via the subcutaneous route. The photomicrograph on the left shows the samples of tissue taken from the striatal quadrants bilaterally.

To address this hypothesis, 5-day-old rat pups (PND5) were injected with a single dose of L-DOPA at either 0 (i.e., vehicle only, n = 13), 25 (n = 14), 50 (n = 12), 75 (n = 14), or 100 mg/kg (n = 15). From 27 to 30 min after the injection, they were allowed to express locomotor movements while suspended in the air (air-stepping [13]) before being sacrificed for CNS extraction. We first report the neurochemical effects of L-DOPA at the level of the cervical spinal cord (cSP) because the analytical conditions allowed us to analyze a large number of compounds of interest. Still, the data were somehow similar in the thoracic (thSP) and lumbar (lSP) spinal cord. Next, we address the dose-dependent effect of L-DOPA administration on the tissue levels of each of the monoamines and their metabolites in sampled brain areas (see Figure 1) with distinct chromatographic conditions than those used for the spinal cord, limiting the number of sampled compounds. We finally report correlative analyses of neurotransmitters and of neurotransmitters with locomotor parameters. A full description of the locomotor effect of L-DOPA on the expression of air-stepping can be found in a previous report [13] and will not be addressed herein.

## 2. Results

### 2.1. Effect of L-DOPA on the Tissue Levels of Monoamines and Associated Compounds in the Cervical Spinal Cord

**Figure 2:**
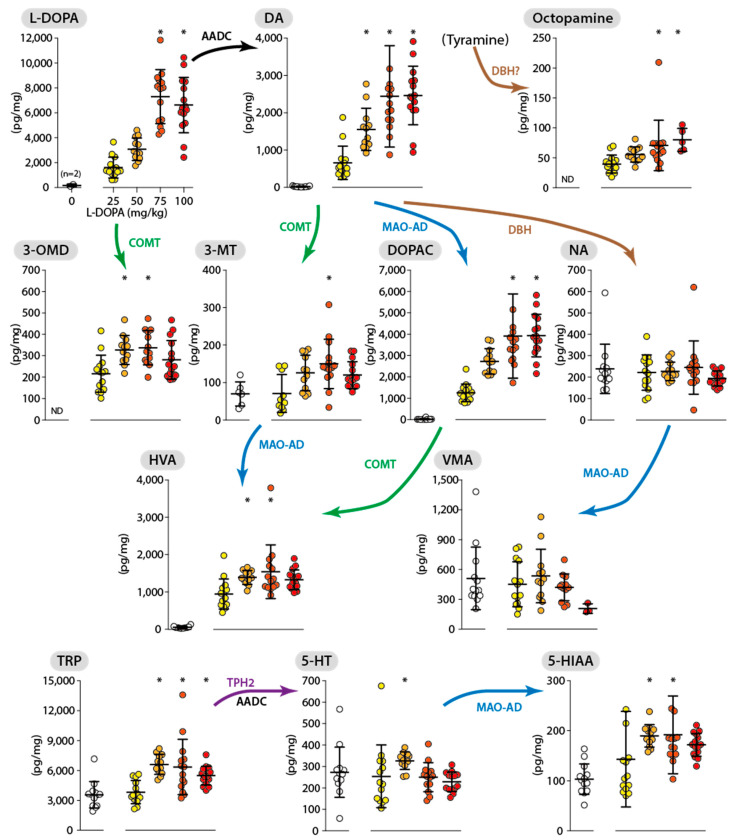
Dose–response effect of L-DOPA on different monoamine-related compounds with their enzymatic link in the cervical spinal cord. Neurochemical measurements are expressed as median/IQR and correspond to the tissue levels obtained 30 min after the injection of L-DOPA at different doses (25, 50, 75, and 100 mg/kg, identified by different colors) or its vehicle. The L-DOPA 25 mg/kg group serves as a reference for the multi-comparison tests (* *p* < 0.05, Dunn’s test). The enzymatic link between compounds has been reported as follows: AADC, aromatic amino acid decarboxylase; DBH, dopamine-β-hydroxylase; COMT, catechol-O-methyl transferase; MAO-AD, monoamine oxidase coupled to aldehyde dehydrogenase; TPH2, tryptophane hydroxylase; 3-OMD, 3-O-methyl L-DOPA; TRP, tryptophan; VMA, vanillylmandelic acid; 3-MT, 3-methoxytyramine. ND, not determined.

The neurochemical effects of L-DOPA in the cervical part of the spinal cord (cSP) are reported in Figure 2 in such a way that the measured compounds are linked to their canonical enzymatic pathway(s). In the cSP, we measured the tissue levels of L-DOPA, its metabolite 3-O-methyl-DOPA (3-OMD), the monoamines dopamine (DA), noradrenaline (NA), and serotonin (5-HT), the metabolites of DA, including 3,4-dihydrophenylacetic acid (DOPAC), homovanillic acid (HVA), and 3-methoxytyramine (3-MT), the precursor and the metabolite of 5-HT tryptophane (TRP) and 5-hydroxyindolacetic acid (5-HIAA), one metabolite of NA, vanillylmandelic acid (VMA), and one product of tyramine, octopamine.

**Figure 3:**
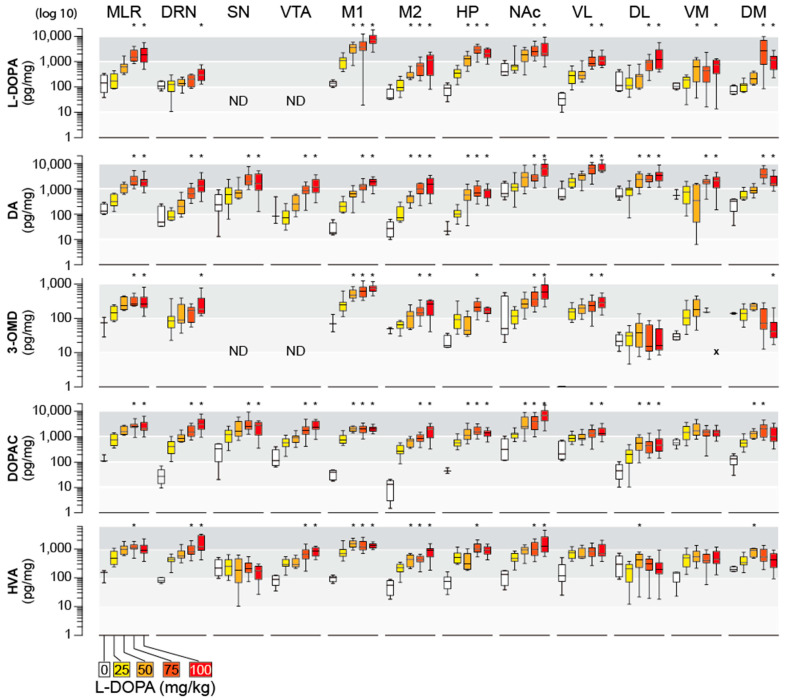
Dose effect of L-DOPA injection on the cerebral levels of L-DOPA, monoamines, and related metabolites. Neurochemical measurements are expressed as median/IQR and were applied on different regions of the brain, which was dissected out 30 min after the injection of L-DOPA at different doses (25, 50, 75, and 100 mg/kg) or its vehicle. M1, M2, motor cortex; NAc, nucleus accumbens; DM, dorsomedial striatum; DL, dorsolateral striatum; VL, ventrolateral striatum; VM, ventromedial striatum; HP, hippocampus; SN, substantia nigra; VTA, ventral tegmental area; MLR, mesencephalic locomotor region; DR, dorsal raphe. The group L-DOPA 25 mg/kg served as reference for the multi-comparison tests (* *p* < 0.05, Dunn’s test; n = 6 pups for vehicle, 12 to 15 for the different doses of L-DOPA). ND, not determined. x: not detected. Note the log10 scale used to better display the disparity of the data distribution between groups and regions.

**Figure 4:**
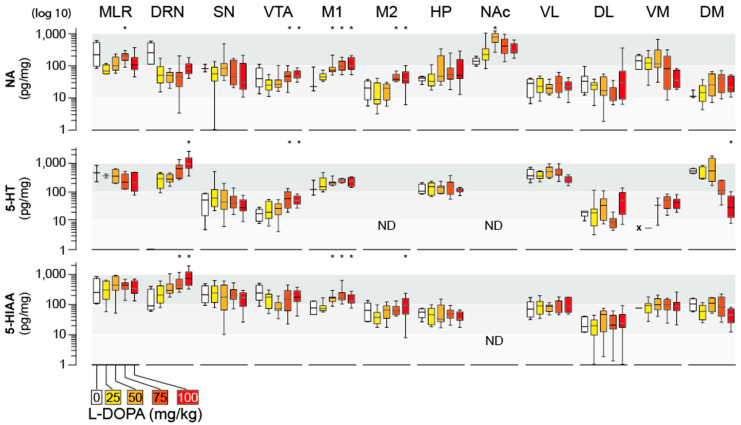
Dose effect of L-DOPA injection on the cerebral levels of NA, 5-HT, and its metabolite 5-HIAA. Neurochemical measurements are expressed as median/IQR and were applied on different regions of the brain, which was dissected out 30 min after the injection of L-DOPA at different doses (25, 50, 75, and 100 mg/kg) or its vehicle. M1, M2, motor cortex; NAc, nucleus accumbens; DM, dorsomedial striatum; DL, dorsolateral striatum; VL, ventrolateral striatum; VM, ventromedial striatum; HP, hippocampus; SN, substantia nigra; VTA, ventral tegmental area; MLR, mesencephalic locomotor region; DR, dorsal raphe. * *p* < 0.05, Dunn’s test. (n = 6 pups for vehicle, 12 to 14 for the different doses of L-DOPA). ND and x, not detected. Note the log10 scale used to better display the disparity of the data distribution between groups and regions.

The basal levels of the different compounds were often low or even below the detection levels (Figure 2). Thus, the basal levels are indicated on figures (also Figure 3 and Figure 4), where possible, but were excluded from the statistical tests (reported in Table 1) so as not to bias the results. As expected, the tissue content of L-DOPA and DA were increased in a dose-dependent manner after L-DOPA administration. In contrast, NA and 5-HT spinal levels were not or were marginally affected by the injection of L-DOPA at different doses. The COMT enzyme was mobilized as a result of the injection of L-DOPA, its activity being highlighted by an increase in 3-OMD, 3-MT, and HVA tissue contents. On the other hand, it appears that the level of VMA is drastically decreased at the highest dose of L-DOPA (100 mg/kg), since it was no longer detectable in 12 of the 15 animals composing the group. The degradation pathway of DA, involving MAO-AD, was also activated by L-DOPA injection, as revealed by the strong increase in DOPAC tissue content, likely explaining in large part the strong increase in HVA. Of note, the products of degradation coming from COMT activity (3-OMD, HVA, and 3-MT) reached their highest levels at the dose of 50 mg/kg L-DOPA, in contrast with DOPAC, the levels of which parallel those of DA. Interestingly, we also found an increase in the cSP content of a compound corresponding to octopamine, with a maximal level obtained for the dose of 100 mg/kg L-DOPA.

Even though L-DOPA did not alter the tissue content of 5-HT in the cSP, it enhanced the tissue content of the 5-HT precursor TRP, as well as the 5-HT metabolite 5-HIAA. The increase in their tissue content (approximately doubled for both of them) already reached a plateau at a dose of 50 mg/kg L-DOPA.

### 2.2. Effect of L-DOPA on Levels of Monoamines and Associated Compounds in the Brain

In response to its administration, L-DOPA accumulated not only in the spinal cord (Figure 2) but also in different brain areas. The highest accumulation was observed in the M1 cortex (Figure 3), but L-DOPA was also elevated in the MLR, NAc, DM striatum and hippocampus. In contrast, L-DOPA less accumulated in the DRN. In contrast, the highest levels of DA were obtained in the striatum (NAc, VL, DL, VM, DM). Of interest, DA was also significantly increased in other motor-related areas, such as the mesencephalic locomotor region (MLR) and motor (M1)/sensory (M2) cortices. The other direct metabolite of L-DOPA, 3-OMD, also exhibited a dose-dependent increase, notably in the MLR, M1/M2, and the striatal NAc and VL (Figure 3), while it remained almost unchanged in the dorsal striatum (DL, DM).

The tissue levels of DOPAC were significantly increased in all CNS regions in the groups receiving L-DOPA, with the highest magnitude of effect observed in the NAc (Figure 3). The only region where DOPAC levels were not significantly affected by the dose of L-DOPA was the dorsolateral (DL) striatum. With the exception of the substantia nigra (SN) and sub-regions of the striatum (VM, VL, DM, DL), where the lowest HVA levels were found, HVA contents were dramatically increased in most CNS regions after L-DOPA administration and, in most cases, in a dose-dependent manner.

The tissue levels of NA were not as much affected in the different brain areas after injection of L-DOPA (Figure 4). An unambiguous dose-dependent increase in NA tissue content was restricted to the M1/M2 cortices and VTA. No modification of tissue content of NA was observed in SN, VL, DM, and DL, as was the case in the spinal cord (Figure 2). In the NAc, we noticed a response corresponding to a U-inverted shape (highest levels observed in the L-DOPA 50 mg/kg group). A slight inhibition, although not significant, was observed in the VM after the injection of the highest dose of L-DOPA (100 mg/kg).

The tissue content of 5-HT and 5-HIAA in response to L-DOPA administration is also reported in Figure 4. From a statistical point of view, the levels of 5-HT were not modified by the administration of L-DOPA in most CNS regions, acknowledging that 5-HT could not be detected in the M2 and NAc, as was the case for the basal levels in the DRN and striatal VM. However, a clear increase in 5-HT content could be highlighted in the DRN and VTA. On the other hand, the 5-HT level in the striatal DM significantly dropped at the highest L-DOPA dose. 5-HIAA tissue content was enhanced by L-DOPA in the DRN and in M1.

### 2.3. Correlative Analyses of Tissue Levels of DA and NA Following L-DOPA Administration

**Figure 5:**
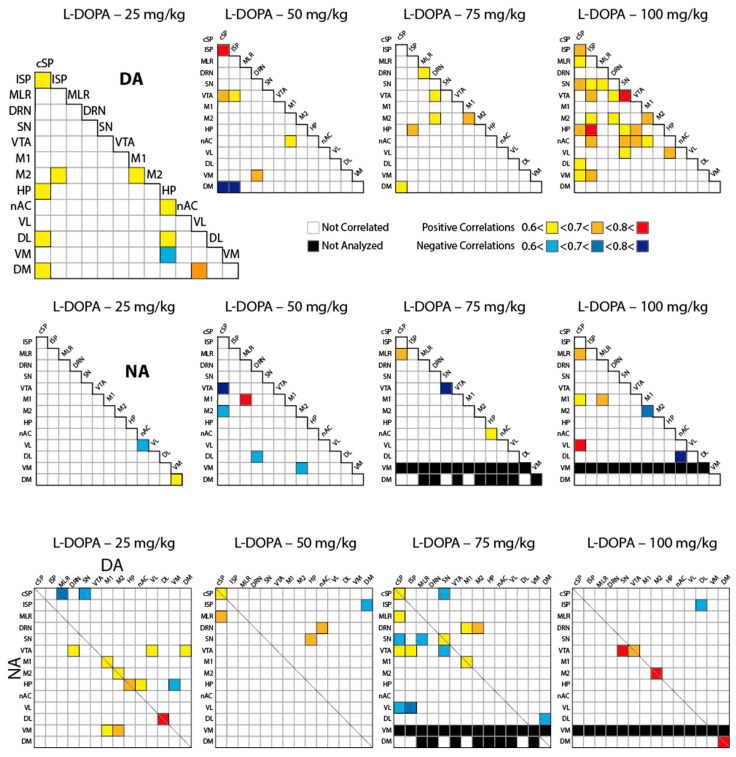
Correlative analysis of the tissue levels of DA, NA, and their combination between the different CNS regions. For each dose of L-DOPA (n = 12 to 14 pups), the tissue levels of DA (**upper panels**), NA (**middle panels**), or their combination DA/NA (**lower panels**) were studied using the Spearman rank order correlation test. Only significant correlations (*p* < 0.05) are reported by the magnitude of the coefficient: positive correlations: <0.6 (yellow) < 0.7 (orange) < 0.8 (red); negative correlations: <0.6 < 0.7 < 0.8 from lighter to darker blue. Black boxes mean that the data were not numerous enough to perform correlative analysis.

The study of correlations of neurotransmitter content across the CNS allows for the study of neurotransmitter connectivity by analogy with studies using functional resonance imaging, possibly highlighting the existence of specific neurobiological networks (patterns of correlations) activated by exogenous L-DOPA injections. In the first analysis, we focused on the inter-regional pattern of correlations established between the tissue levels of DA and NA, given that L-DOPA is the precursor of their synthesis (Figure 5). 5-HT was excluded because it was below the detection threshold in some regions and samples.

Considering the CNS levels of DA, correlation analysis revealed that increasing the dose of L-DOPA to 100 mg/kg enhanced the overall number of inter-regional correlations (to a total of 91 inter-regional correlations) compared to the lower doses investigated (Figure 5, upper panels; L-DOPA 25 mg/kg: 10/91 correlations; 50 mg/kg: 7/91; 75 mg/kg: 6/91; 100 mg/kg: 23/91). Of note, all inter-regional correlations were positive following the injection of 100 mg/kg L-DOPA. However, we noticed that the pairs of regions that exhibited a correlation between their DA tissue content were not the same from one L-DOPA condition to another. The number of correlations of inter-regional NA levels remained lower than for DA (Figure 5, middle panels). There was a positive correlation between NA content in the MLR and cSP reported at 75 and 100 mg/kg L-DOPA. cSP and M1 displayed the highest number of correlations.

When combined, the correlations tended to be concentrated in telencephalic regions at 25 mg/kg L-DOPA and in brainstem/spinal cord regions at 75 mg/kg L-DOPA. We noticed that the highest number of correlations between DA and NA was seen at 75 mg/kg L-DOPA, a dose at which DA showed the lowest inter-regional correlation number. Overall, the DA/NA correlations did not show a clear, dose-dependent spatial pattern of accumulation across the CNS.

**Figure 6:**
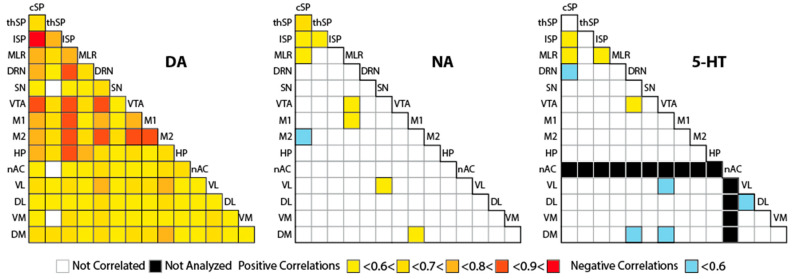
Correlative analysis of the tissue levels of DA, NA, and their combination across the sampled CNS regions. For each dose of L-DOPA (n = 12 to 14 pups), the tissue levels of DA (**upper panels**), NA (**middle panels**), or their combination DA/NA (**lower panels**) were studied using the Spearman rank order correlation test by pairs of brain regions. Only significant correlations (*p* < 0.05) are reported by the magnitude of the coefficient: positive correlations: <0.6 (yellow) < 0.7 (orange) < 0.8 (red); negative correlations: <0.6, blue. Gray boxes mean that the data were not numerous enough to perform correlative analysis.

Pooling the data obtained at the different doses of L-DOPA from 25 to 100 mg/kg, nonetheless, revealed two different profiles between the correlations established for DA versus NA and 5-HT (for which the total number of values allowed for such analysis; Figure 6). In the case of DA, we can easily establish that there is a global increase in DA content, with correlations between virtually all CNS regions. It seems that DA levels is a result of a ubiquitous metabolic response to L-DOPA injections, as anticipated from the quantitative analyses (Figure 2 and Figure 3). Of note, the most prominent correlation was observed between the cervical and lumbar spinal cord, which is where the locomotor networks are located. The correlation maps (Figure 6) for NA and 5-HT contrast in terms of the number of positive correlations, which remained restricted mostly to the spinal cord and the mesencephalic locomotor region (MLR), key areas in locomotor control. 5-HT content displayed negative correlations in the striatum or the DRN.

### 2.4. Correlations Between Neurochemical Markers and Behavioral Parameters

**Figure 7:**
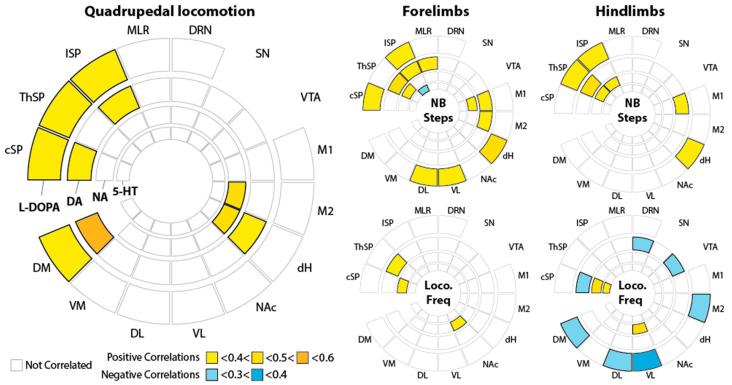
Correlative analysis of the tissue content of compounds and kinematic parameters stimulated by L-DOPA. The figure reports the coefficient of correlation of Spearman for compounds and kinematic parameters. The correlations were performed irrespective of the administered dose of L-DOPA. The kinematic parameters were as follows: % of quadrupedal activity, frequency of forelimb (Freq FL) and hindlimb (Freq HL) locomotor movements, and the corresponding number of cycles (NB steps FL, NB steps HL).

As a final step, we addressed whether correlations between CNS monoamines and the locomotor activity monitored 30 min after L-DOPA injections could be revealed (Figure 7). Of note, none of the animals that did not walk (all animals receiving the vehicle and mostly all animals receiving 25 mg/kg L-DOPA) were included in this analysis. The locomotor parameters were extracted from the sequences of air-stepping expressed during the 3 min period preceding collection of the brain and spinal cord. These included the % of quadrupedal locomotion, the number of forelimb and hindlimb steps, as well as the forelimb and hindlimb stepping frequencies reported in our previous publication [13].

These analyses revealed that the spinal cord levels of L-DOPA, DA, and, less predominantly, NA positively correlate with the global level of quadrupedal locomotion (i.e., the percentage of time all four limbs are stepping) and the associated number of steps at both the forelimb and hindlimb levels (Figure 7). We also noticed a correlation of M1 DA levels with the number of steps. Interestingly, more positive correlations could be revealed between the brain areas and the forelimb number of steps in comparison to the hindlimbs. Few positive correlations could be established between monoamine levels across the CNS and the frequency (speed) of the locomotor movements.

## 3. Discussion

We report a dose–response effect of exogenously administered L-DOPA on the tissue content of monoamines and derivatives in various CNS regions of neonatal rats that exhibited episodes of air-stepping in response to L-DOPA administration. The neurochemical responses to L-DOPA show a solid and homogenous response to DA and DOPAC tissue content across the CNS. Responses were far more variable across regions for NA and 5-HT tissue content following L-DOPA administration. Similarly, the correlative link between tissue content and locomotor parameters was stronger for L-DOPA and DA than for NA, and even more so than 5-HT. As far as DA is concerned in the locomotor effects induced by L-DOPA, our data indicate that some of the sampled CNS regions cannot be excluded from its mechanism of action but suggest a prominent influence of mechanisms within the spinal cord and to some extent to the mesencephalic locomotor region and sensori-motor cortices.

The induction of air-stepping in pups by L-DOPA is an intriguing response that involves the spinal cord [13,14,15], without excluding the possibility of a contribution of the supraspinal centers. From a biochemical point of view, the injection of L-DOPA (25–100 mg/kg, ip) dose-dependently enhanced L-DOPA tissue content in all sampled CNS regions from the cortex to the spinal cord. It is noteworthy that the injections of L-DOPA occurred without a peripheral AADC, inhibitor which is supposed to favor above five to ten times the entry of L-DOPA in the CNS of adults [5]. The accumulation of L-DOPA differed between CNS regions. It is interesting to note that the highest accumulation occurred in the spinal cord and M1. Several factors can be proposed to account for this, but it likely depends on the enzymatic material available in the distinct brain regions. Several enzymes catabolize L-DOPA, and we considered the aromatic amino acid decarboxylase (AADC) and the catechol-o-methyl transferase (COMT). As regards the AADC, the production of DA from exogenous L-DOPA was tremendous in all CNS regions, but the strongest responses were observed in striatal quadrants. This strong and expected activity in the striatum is likely due to high expression of AADC in DA terminals and striatal neurons. Of note, at PND5, monoaminergic neurons including DA neurons did not finish their maturation, which is supposed to occur beyond PND30 [26,27]. The basal values for DA and DA metabolites, as well as for 5-HT and NA, in the brain were lower compared to adult rats [28,29]. Our results also show that, in most structures, COMT is very active in metabolizing L-DOPA into 3-OMD.

DA is also metabolized by the monoamine oxidase (MAO) coupled to aldehyde-dehydrogenase (AD) to give DOPAC and by COMT to give 3-MT as we report in the spinal cord [22,30]. MAO is the main catabolic pathway for DA in the newborn rat, as revealed by the substantial amounts of DOPAC measured in all CNS regions. The tissue levels of HVA, which involves both COMT and MAO, were also dramatically increased in several regions, although this activity was less prominent in the striatum and the SN. Thus, COMT and MAO/AD enzymes are present in all regions, where they work synergistically for the degradation of L-DOPA and DA. Yet, regional differences in the COMT and MAO/AD activity ratio may explain some regional disparity in the tissue content of L-DOPA, DA, and their metabolites.

Among them, we were able to measure octopamine in the spinal cord in response to L-DOPA [22], although we could not keep those conditions for the analyses of brain regions (see methods). The presence of octopamine could imply an increase in tyramine in the tissue. Speculatively, the presence of L-DOPA may suppress the activity of tyrosine hydroxylase, indirectly favoring the decarboxylation of tyrosine into tyramine. The presence of tyramine (and octopamine) in the CNS after L-DOPA injection might be a confounding factor altering our understanding of the distribution of tissue monoamines including NA and 5-HT and their release. Indeed, tyramine is even more powerful than DA in substituting the other monoamines from their storage vesicles [22]. Meanwhile, the effects of L-DOPA on tissue content of NA and 5-HT are not intuitive because they are region-dependent and away from classical views.

Even though L-DOPA is a metabolic precursor of NA, our results show that its exogenous administration does not lead to a massive and systematic CNS accumulation of NA. In fact, NA accumulations were restricted to VTA, M1, and M2. Even the highest response we measured in the NAc after medium doses of L-DOPA disappeared after higher doses. The lack of NA accumulation could be primarily due to region-specific differences in Dopamine-Beta-Hydroxylase (DBH) availability/activity. Concurrently, an increase in DA inside NA neurons and an entry of DA (also tyramine or octopamine) inside the vesicles of exocytosis could also account for a lack of NA accumulation, as it is believed that L-DOPA-derived DA chases NA from the storage vesicles at least at high doses of L-DOPA (50 mg/kg + peripheral AADC inhibitor) [23]. Alternatively, regional differences in the catabolic chain degrading NA could also contribute to our observations. Here, we were only able to measure the tissue levels of VMA, one of the NA metabolites [30], in the spinal cord. There is no evidence that the metabolic pathway leading to the formation of VMA is responsible for the stable levels of NA.

For the 5-HT system, we extend previous data in the lumbar spinal cord to 5-HT levels in the cervical spinal cord and the majority of the brain regions investigated to conclude that exogenous L-DOPA does not affect the central levels of 5-HT [13,18]. Of interest, the only two regions where 5-HT accumulates significantly post L-DOPA are the VTA and the DRN. It is known that L-DOPA-derived DA substitutes 5-HT from storage sites [24,25,31,32,33,34]. While an increase in 5-HIAA levels could be expected, it was not clearly reported in most brain regions, with the exception of the DRN, M1, and spinal cord. The decrease in 5-HIAA levels, as seen in the DM, could also be associated with the chase of 5-HT induced by DA toward the extracellular space [5]. The determination of 5-HT was complicated in some brain regions, including the NAc, VM, M2, and DRN. The simplest explanation would be that 5-HT levels were below the detection levels in these areas. We cannot exclude technical issues, for instance, the precipitation of 5-HT, especially in a context where 5-HIAA was concomitantly detected (except in the NAc). Alternatively, in two brain regions, 5-HT was, however, detected upon the administration of L-DOPA, particularly in the DRN, suggesting that L-DOPA (perhaps via DA) promotes a reorganization of the 5-HT pools inside 5-HT neurons. Further work will be needed to confirm the status of 5-HT in these brain regions, especially in basal conditions, which are perhaps linked to poor though distinct maturity of the tissue among individuals.

The connectivity of DA and NA could inform the organization of neurochemical responses to L-DOPA between territories [28]. At the highest dose, correlative links involved the spinal cord (notably cSP), mesencephalon (notably SN, VTA), hippocampus, and some striatal quadrants for DA and cSP, M1, MLR, or VL for NA. The responses were not progressive with respect to the doses of L-DOPA. One explanation is that the other monoamines like NA interact with DA at different levels. For instance, the lowest number of inter-regional correlations for DA was reported at 75 mg/kg L-DOPA, which corresponded to the highest level of correlations between DA and NA across the CNS. When pooling all L-DOPA conditions, impressive levels of correlations for DA were indeed reported. This analysis also revealed a few numbers of inter-regional correlations for 5-HT and NA, highlighting the spinal cord and the MLR.

Acknowledging that exogenous L-DOPA can trigger several effects beyond its conversion into DA [5,35], the correlations of neurochemical parameters with behavior still suggest that DA plays a major role in L-DOPA’s locomotor action. The locomotor/behavioral responses to L-DOPA in pups are an interesting paradigm to study the modulatory roles of monoamines at early stages [16,17]. Our neurochemical data show a clear, powerful DA response at the level of spinal cord segments. It is in line with previous data causally addressing the role of spinal cord/brainstem in air-stepping responses to L-DOPA using electromyographic measurements or kinematic analyses [10,15,36]. This is consistent with the fact that L-DOPA-induced air-stepping has been shown to be reduced by an AADC inhibitor [19], the AADC competitor 5-HTP [13], and non-selective DA antagonists [14] and potentiated by the COMT inhibitor tolcapone or the MAO inhibitor nialamide [18]. The hindlimb locomotor generator is located in the lumbar spinal cord [37], where we showed that L-DOPA pro-locomotor action was concomitantly associated with a dose-dependent increase in DA, but not 5-HT [13]. Here, we could extend these observations to the cervical spinal cord, where the locomotor neural network commanding the forelimbs is located [38]. As regards the CNS, the correlation of locomotor activity with CNS monoamines first suggested that the speed of the locomotor movements seems less or non-dependent on CNS monoamine levels. In contrast, at this developmental stage, spinal DA appeared to be strongly correlated with the expression of a quadrupedal locomotor activity, especially at the levels of the cervical and lumbar locomotor networks. This observation could also be established while considering the number of forelimb and hindlimb steps with the spinal catecholamine levels. Interestingly, one main difference between the forelimbs and hindlimbs was that more brain regions correlated positively with the number of forelimb steps. This difference is likely explained by the progressive maturation of the monoaminergic descending pathways in the spinal cord, with a lumbar innervation occurring at later stages compared to the cervical cord [39,40,41,42].

Higher levels of L-DOPA accumulation have recently been reported in extrastriatal regions in monkeys exhibiting L-DOPA-induced dyskinesia [43]. Different works have highlighted the role of motor cortex in adult rats [44,45] and parkinsonian patients [46,47] in L-DOPA-induced dyskinesia. Our result suggests that the spinal cord could also contribute to L-DOPA related motor disorders. The possible involvement of the DA and 5-HIAA DRN content in behaviors is not surprising because 5-HT neurons are the primary neuronal system involved in the release of DA induced by L-DOPA [24,25,48,49]. While the DRN innervates the encephalon, it is likely that the descending 5-HT neurons from the caudal raphe nuclei function similarly to promote L-DOPA-derived DA release in the spinal cord of newborn rats [13].

These mechanisms are likely underestimated in the mechanism of action of L-DOPA in Parkinson’s disease. Indeed, the air-stepping response is lost after PND20 [15], but the neurochemical responses should not disappear. The increase in tryptophan tissue content induced by exogenous L-DOPA (Figure 2) recalls that L-DOPA enters cells via the aromatic amino acid transporter in most cells and neurons [5]. Its decarboxylation into DA is simply due to AADC activity which is present in pups and adults. Thus, the impact of L-DOPA on locomotion, presumably via its decarboxylation into DA and involving the modulatory actions of both 5-HT and NA systems [13,50,51], is dependent on the maturation of the neurobiological networks. This idea was previously addressed by showing that the c-Fos response in the brainstem and basal ganglia (mostly the subthalamic nucleus and SN) induced by L-DOPA was very low at PND25 (no air-stepping) compared to PND15 (air-stepping) [21]. Considering that L-DOPA-induced DA release is relatively low in the striatum compared to extra-striatal tissues in DA neuron-lesioned adult rats [5], the motor benefits of L-DOPA in Parkinson’s disease could correspond to those observed in immature tissue as PND5: it could involve M1, parts of the brainstem, and spinal cord as regional components. Additional studies are warranted to describe such a possibility.

## 4. Materials and Methods

### 4.1. Animals

A total of 68 Sprague Dawley newborn rats (postnatal day 5, PND5) of either sex and bred in the laboratory were used in the present study. All procedures were conducted in accordance with the local ethics committee of the University of Bordeaux, followed the European Committee Council directives (2010/63/EU), and were validated by the French Ministry for Higher Education, Research and Innovation (APAFIS #11978). The number of animals used and animal suffering were reduced to a strict minimum. Animals were kept in the animal facility with their mother prior to their manipulation (PND5). After treatment injection, they were placed in the air-stepping device in a room kept at 28 °C for 30 min. Individuals were ascribed to only one experimental pharmacological condition. As previously described [13], the behavioral sequences of air-stepping were recorded for 3 min, 30 min after the drug’s injection, corresponding to the drug’s maximal effect [14,15].

### 4.2. Pharmacology

On the day of the experiment, 50 mg of L-3,4-dihydroxyphenylalanine (L-DOPA, ab120573, Abcam Biochemicals) was prepared at a dose of 10 mg/mL in HCl (1N, 350 µL), distilled water (150 µL), and Na_2_HPO_4_ (4.5 mL). The solution was stirred to obtain a clear homogenous injectable preparation. Control animals received the equivalent volume of the vehicle solution. In all cases, the solution was injected subcutaneously into the nape of the animal using a 30G needle. The volume injected was adjusted to the weight of each animal, within the range of 2.5–15 µL/g, at doses of 0, 25, 50, 75, and 100 mg/kg. The doses were selected on the basis of previous studies [13,15].

### 4.3. Air-Stepping

The whole procedure was recently published in detail [13]. Briefly, reflective markers (diam 3 mm, optitrack) were stuck bilaterally on the hip, scapula, and toes of the fore- and hindlimbs. After drug injection, animals were suspended in the air in a harness [13,18]. Lights placed on both side of the animals were used to illuminate the markers during the recording. Two synchronized microcomputer-controlled cameras (https://www.raspberrypi.org) were used to record the sequences of air-stepping bilaterally (frame rate of 49 Hz). Video files of 3 min duration were stored on a computer and analyzed off-line using Kinovea software (version 0.9.5, Kinovea.org). The locomotor movements were extracted as angular excursions of all limbs calculated from the x-y coordinates of the markers, which were detected on an image-by-image basis. Next, limb excursions were used to calculate the different locomotor parameters using routines written in Matlab (version R2015b, Mathworks, Natick, MA, USA), including the step cycle frequency and the step amplitude (corresponding to the step length in overground locomotion).

### 4.4. Collection of Samples and Tissue Conditioning

Thirty minutes after L-DOPA or vehicle injection, and immediately after video acquisition, animals were sacrificed, and the brain and spinal cord were rapidly dissected out and frozen in isopentane (approximately −35 °C). The cervical (cSP) and lumbar (lSP) spinal cord regions were placed in pre-weighed tubes (0.6 mL) and, together with the brains, were stored at −80 °C. Pup brains were subsequently placed in a cryostat (−24 °C) to take out, using a stainless-steel punch, the following 12 regions: a part corresponding to the mesencephalic locomotor region (MLR), the dorsal raphe nucleus (DR), the substantia nigra (SN), the ventral tegmental area (VTA), the dorsal part of the hippocampus (HP), motor cortices M1 and M2, and 5 striatal regions including the nucleus accumbens (NAC), the dorsolateral (DL), dorsomedial (DM), ventromedial (VM), and ventrolateral (VL) striatum (Figure 1). The bilateral parts of the sampled regions were placed together in a pre-weighed tube (0.6 mL). All tissues were rapidly brought to deep freezer (−80 °C) until neurochemical analysis. On the day of biochemical analysis, tubes containing the tissue were placed on ice and rapidly wiped and weighed using the same balance. Samples were then homogenized in 0.1N HClO_4_ (100 µL), sonicated, and centrifuged at 13,000 rpm for 30 min at 4 °C. The supernatant (10 or 20 µL depending on the region) was injected into high-performance liquid chromatography (HPLC) systems coupled to electrochemical detection.

### 4.5. Neurochemical Analysis

In most cases, we measured the tissue levels of dopamine (DA), noradrenaline (NA), and serotonin (5-HT)), L-DOPA, and some of their metabolites, including 3-O-methyl-DOPA (3-OMD), 3,4-dihydrophenylacetic acid (DOPAC), homovanillic acid (HVA), and 5-hydroxyindolacetic acid (5-HIAA) [28]. Unfortunately, while ensuring that one CNS region was studied using the same HPLC system, we could not always reproduce identical conditions over the whole study (see below).

In all cases, the HPLC system comprised a manual injector (Rheodyne 7725i, C.I.L. Cluzeau, Sainte-Foy-La-Grande, France) equipped with a 20 µL loop, a HPLC column (Hypersyl C18, 150 × 4.6 mm, 5 µm, C.I.L. Cluzeau) preceded by a Brownlee-Newgard precolumn (RP-8, 15 × 32 mm, 7 µm, C.I.L. Cluzeau), an HPLC pump (either LC-20-AD or LC10 AD vp Shimadzu, France, or GOLD 128, Beckman, France), one or two electrochemical cells (5011 coulometric cell, ESA, Paris, France), one or two programmable detectors (Coulochem II, ESA, Paris, France) [22], and an Ulyss interface coupled to a computer using dedicated software (Azur 5.0, Datalys, Saint-Martin-d’Hères, France). The mobile phase was prepared in deionized water and composed of 60 mM NaH_2_PO_4_, 0.1 mM Disodium EDTA, containing 7% methanol filtered (0.22 µm Millipore filter). The mobile phase was delivered at 1.2 mL/min. The concentration of the pairing ion 2-Octane-Sulfonic acid (approximately 150 mg/L) and the pH (usually around 4) were adjusted to obtain appropriate conditions of eluent separation/sensitivity in the chromatogram. This was performed while also considering the elution time of other compounds that can be oxidized, such as vanillylmandelic acid (VMA), 3-methoxytyramine (3-MT), 5-hydroxytryptophan, dihydroxyphenylethylene glycol, 3-methoxy-4-hydroxy ethyleneglycol, octopamine, tyrosine, and tryptophan (TRP). In its simplest configuration (one coulometric cell with the potential of the electrodes set to −270 and +350 mV coupled to one detector), our system could manage the oxidation of the main compounds of interest (used for SN, VM striatum, and VTA) with a correct separation of 5-HT and 3-MT, but an error in the calibration curve for L-DOPA and 3-OMD discarded these compounds in some of the samples corresponding to these brain regions. In another system, we coupled two cells in series whose potentials were set at −100 and +350 mV, +450 and +580 mV (used for the 3 spinal parts and DL). These conditions were good, as 5-HT and 3-MT were separated, while the different potentials allowed us to also measure VMA, octopamine, and TRP [22]. The last conditions were conditioned by our impossibility to correctly separate 3-MT and 5-HT. In that case, we chose to fix the 4 potentials in series at 50 mV, 180 mV (5-HT releases electrons but 3-MT does not at this potential), −100 mV, and 350 mV (used for MLR, M1, M2, NAC, HP, VL, and DM). Calibration curves were obtained using standard solutions at three distinct and known concentrations for each compound of interest. Moreover, a standard solution (1 ng/10 µL) was administered before and after each daily series of sample analysis.

### 4.6. Statistics

Neurochemical data were analyzed using SigmaPlot. Descriptive statistics are presented as median/IQR (interquartile range). Comparison of the neurochemical data between groups was achieved using a Kruskall–Wallis test followed by a Dunn’s multiple comparisons test. In all cases, data collected in the experimental group receiving the vehicle solution without L-DOPA were provided but not included so as not to bias the statistics between the groups receiving L-DOPA at different doses. Data from the group L-DOPA 25 mg/kg were taken as reference for the multiple comparisons test. Correlations of neurochemical data between CNS regions were studied by the Spearman correlation test. Similarly, correlations between neurochemical and behavioral data were analyzed by Spearman correlation tests, acknowledging that only animals exhibiting air-stepping in response to L-DOPA were included in this later analysis. Statistical significance was set at *p* < 0.05.

## 5. Conclusions

The study shows that exogenous L-DOPA dose-dependently enhances the tissue content of 3-OMD, DA, and its related metabolites in the forebrain, brainstem, and spinal cord regions of newborn rats (PND5). It probably enhances other tyrosine-related chemical species such as octopamine, at least in the spinal cord. The effects of L-DOPA on NA and 5-HT function are low and/or unequal between regions. However, the extended spatial pattern of action of L-DOPA, observed as early as the postnatal stage, could account to some extent to the motor responses notably observed in the parkinsonian state in response to L-DOPA treatment.

## Figures and Tables

**Table 1:** Statistical report of the effect of L-DOPA on tissue levels of the various compounds in the different CNS regions.

	H (Kruskall–Wallis)							
Regions	L-DOPA	DA	3-OMD	DOPAC	HVA	NA	5-HT	5-HIAA
cSP	**39.78**	**31.34**	**19.70**	**53.19**	**35.54**	4.67	**14.40**	**28.59**
thSP	**28.** **19**	**29.23**	**6.33**	**29.43**	**26.69**	2.19	**10.11**	**20.62**
lSP	**41.63**	**38.21**	**22.59**	**36.03**	**19.95**	5.75	**11.48**	**17.97**
MLR	**25.79**	**21.62**	**10.57**	**17.59**	**10.07**	**9.58**	2.47	2.31
DRN	**12.94**	**33.84**	**11.69**	**32.80**	**23.30**	5.72	**21.36**	**18.79**
SN	/	**19.37**	/	**12.88**	3.03	2.76	4.14	3.03
VTA	/	**32.30**	/	**29.43**	**25.60**	**16.20**	**13.94**	**7.93**
M1	**31.37**	**35.29**	**26.77**	**24.92**	**15.69**	**22.45**	2.096	**22.03**
M2	**23.81**	**33.47**	**18.17**	**28.87**	**23.82**	**19.** **84**	/	6.70
dH	**34.69**	**27.89**	**24.87**	**22.90**	**21.83**	4.20	2.06	1.66
NAc	**19.30**	**19.35**	**26.73**	**18.96**	**16.59**	**13.81**	/	/
VL	**31.29**	**30.13**	**11.92**	**10.55**	1.49	0.92	7.35	0.67
DL	**29.58**	**22.91**	1.51	**13.63**	6.96	5.04	**15.97**	5.07
VM	**10.65**	**20.02**	3.04	1.66	1.71	**8.** **16**	/	0.51
DM	**24.49**	**29.06**	**16.23**	**17.16**	**9.43**	5.12	**22.64**	7.05

Additional statistics (H of Kruskall–Wallis, all significant whenever available) in cSP, thSP, and lSP, respectively, for TRP [**31**, **22**, **25**], 3-MT [**15**, **8**, **15**], VMA [**33**, **31**, **31**], and octopamine [**18**, not determined, **24**]. lSP, lumbar spinal cord; thSP, thoracic spinal cord; cSP, cervical spinal cord; MLR, mesencephalic locomotor region; DRN, dorsal raphe nucleus; SN, substantia nigra; VTA, ventral tegmental area; M1 and M2, motor cortices; dH, dorsal hippocampus; NAc, nucleus accumbens; VL, ventrolateral striatum; DL, dorsolateral striatum; VM, ventromedial striatum; DM, dorsomedial striatum. The values of H in bold indicate that *p* < 0.05; **/**: not determined. Number of pups/group: 25 mg/kg: 14; 50 mg/kg: 12; 75 mg/kg: 14; 100 mg/kg: 15.

## Data Availability

Database is available upon kind request.
